# Cardiovascular risk factors in Assyrians/Syrians and native Swedes with type 2 diabetes: a population-based epidemiological study

**DOI:** 10.1186/1475-2840-8-59

**Published:** 2009-11-12

**Authors:** Marina Taloyan, Alexandre Wajngot, Sven-Erik Johansson, Jonas Tovi, Jan Sundquist

**Affiliations:** 1 Center for Primary Health Care Research, Region Skåne, Lund University, Sweden, UMAS, 205 02 Malmö, Sweden; 2 Karolinska Institutet, Center for Family and Community Medicine, Sweden, Alfred Nobels allé 12, SE -141 83 Huddinge, Sweden; 3 Stanford Prevention Research Center, Stanford University School of Medicine, California, USA

## Abstract

**Background:**

A large number of people throughout the world have diabetes and the prevalence is increasing. Persons with diabetes have a twice higher risk of cardiovascular disease than those without diabetes. There is a lack of studies focusing on cardiovascular risk factors in Assyrians/Syrians with type 2 diabetes. The aim of this study is to estimate the prevalence of some cardiovascular risk factors among Assyrians/Syrians and native Swedes with type 2 diabetes and to study whether the association between ethnicity and cardio-vascular risk factors remains after adjustment for age, gender, employment status and housing tenure.

**Methods:**

In the Swedish town of Södertälje 173 Assyrians/Syrians and 181 ethnic Swedes with type 2 diabetes participated in a study evaluating cardiovascular risk factors such as increased haemoglobin A1c (HbA1c), high blood lipids (total serum cholesterol and triglycerides), hypertension and high urinary albumin. The associations between the outcome variables and sociodemographic characteristics were estimated using unconditional logistic regression.

**Results:**

The prevalence of increased triglycerides in Swedish-born subjects and Assyrian-Syrians was 61.5% and 39.7% respectively. Swedes had a prevalence of hypertension 76.8% compared to 57.8% in Assyrians/Syrians. In the final logistic models adjusted for gender, age, housing and employment the odds ratio (OR) for Swedish-born subjects for increased triglycerides was 2.80 (95% CI1.61-4.87) and for hypertension 2.32 (95% CI 1.35-4.00) compared to Assyrians-Syrians.

**Conclusion:**

Ethnic Swedes had higher prevalence of increased triglycerides and hypertension than Assyrians/Syrians. Total cholesterol, HbA1c and urinary albumin did not differ between the two ethnic groups.

## Background

Type 2 diabetes is a growing public health issue throughout the world that has a huge impact on society in terms of health care costs and individual suffering. For example, type 2 diabetes increases the risk of cardiovascular complications and at present more than 180 million people worldwide have diabetes [1]. The presence of type 2 diabetes doubles the risk of cardiovascular disease compared to people without diabetes [2]. Thus, approximately 1.1 million people died of diabetes in 2005, mainly because of cardiovascular complications [1].

In Sweden, about 12% of the population is born abroad and 41% originate from outside of Europe, with 15% from the Middle East [3]. The Swedish population of Assyrians/Syrians, about 20 000 persons, originates from Turkey, Syria, Iraq and Lebanon. Most of them live in Södertälje where first and second generation immigrants constitute 36% of the total population. *Assyrian *is the definition of groups in the Middle East who belong to one of the following four churches - the Syrian-orthodox, the Nestorians, the Caldeian and the Syrian-catholic. *Syrian *is the definition of the Syrian-orthodox group and those who don't want to be defined as Assyrian [4]. Thus, there are two ways of self-identification among the studied immigrant population we chose to regard them as one ethnic group with two identification definitions - Assyrians/Syrians.

The prevalence of diabetes, mainly type 2, is about 4% in Sweden [5]. A Swedish study from primary health care centres in Stockholm County showed that the age and gender-standardised prevalences of diabetes among native Swedish patients aged 35-64 were 1.80% and 5.82% and were three times higher in patients from non-European countries [6]. According to several studies, stress associated with urbanisation and changes in lifestyle contributes to the higher prevalence of type 2 diabetes among patients from non-European countries [7,8]. A recently published study on comparing clinical characteristics of type 2 diabetes between immigrants from the Middle East and Swedish-born patients shows that immigrants from the Middle East have more marked family history, an earlier onset and a different form of type 2 diabetes compared to Swedish-born patients [9].

Despite the large number of immigrants in Sweden there is a lack of knowledge about diabetes in this group of people. In general, immigrants from the Middle East have a poorer perception of health than native Swedes [10]. They develop their type 2 diabetes earlier in life and they are more overweight. This might be explained by factors pertaining to migration per se as well as to such lifestyle factors after migration as low levels of employment and education and higher prevalence of overweight and obesity among immigrants [11].

There are also important ethnic differences concerning cardiovascular disease and risk factors when comparing immigrants from Iran and Turkey to the native Swedish population in general. The higher incidence of diabetes and cardiovascular risk factors and disease in immigrants in comparison to Swedish-born subjects is attributed by several researchers to a higher prevalence of abdominal obesity and higher levels of lipids [12] and to lifestyle factors [13]. The opposite results were observed in a study in patients with type 2 diabetes between Turkish and Dutch groups in the Netherlands with similar absolute risk for a coronary event [14].

There is a lack of knowledge about type 2 diabetes in Assyrian/Syrian patients and their cardiovascular risk factors. We therefore address this issue in this study and compare cardiovascular risk factors between ethnic Assyrians/Syrians and Swedes with type 2 diabetes living in the same area. The first aim is to estimate the prevalence of some cardiovascular risk factors among Assyrians/Syrians and native Swedes with type 2 diabetes and, secondly, to study whether the association between ethnicity and cardiovascular risk factors remains after adjusting for age, gender, employment status and housing tenure.

## Methods

### Participants

A total of 354 individuals with type 2 diabetes were included: 173 Assyrians/Syrians and 181 native Swedes. The study was conducted during 2006-2008. Participants were found in the register of diabetes patients at the four primary health care centres in the town of Södertälje. They were interviewed face-to-face by the first author and different GPs, with (if needed) and without interpreters, at the primary health care centres. The participants were Assyrians/Syrians and a gender- and age-matched group of native Swedes. The countries of origin of the Assyrians/Syrians were all Middle Eastern, i.e. Turkey (33.5%), Iraq (30.6%), Syria (20.2%) and the remaining group originated from Lebanon or other countries. Medical information and laboratory data from patient charts were gathered after obtaining the verbal informed consent of all participants. Blood pressure was measured in the sitting position following 5 minutes of rest after the interview.

### Outcome variables

*HbA1c *was divided into two groups: normal (≤ 6.0%) and high (> 6.0%) (Swedish mono-S method [15]).

#### Cardiovascular risk factors

*Total cholesterol *was divided into two groups: normal (< 4.5 mmol/L) and high (≥ 4.5 mmol/L).

*Triglycerides *were divided into normal (< 1.7 mmol/L) and abnormal (≥ 1.7 mmol/L).

#### Hypertension

*Systolic blood *pressure was dichotomised as normal (≤ 130 mmHg) and high (> 130 mmHg).

*Diastolic blood *pressure was divided into normal (≤ 80 mmHg) and high (> 80 mmHg).

If either the systolic blood pressure was > 130 mmHg and/or the diastolic blood pressure was > 80 mmHg, the patient was considered to be hypertensive, otherwise non-hypertensive.

*Urinary albumin *levels were divided into normal (< 3.4 mg/mmol/L) and non-normal (≥ 3.4 mg/mmol/L).

#### Explanatory variables

*Age *was divided into three groups: 32-57, 58-70 and >70 years.

*Ethnicity *was defined as native Swedish and Assyrian/Syrian immigrants with both first and second generations of immigrants.

*Employment status *during the preceding week consisted of two alternatives: (1) employed (including any type of employment, sick leave and vacation) and (2) unemployed (including unemployment, retirement and studies).

*Housing tenure *was defined as ownership or rental.

### Statistical analyses

We estimated the prevalence of the outcome variable for both ethnic Assyrian/Syrian and Swedish subjects separately using the statistical software program Stata v.9 [16]. Pearson's chi^2 ^test and t-test were used to test the level of significance in the prevalence of high HbA1c, increased blood lipids (total cholesterol, triglyceride), hypertension and increased urinary albumin. Unconditional logistic regression was used to estimate the odds ratios (ORs) and 95% confidence intervals (95% CIs) to analyse the association between cardiovascular risk factors and independent variables. We present the final model including all explanatory significant variables: age, gender, ethnicity, employment status and housing tenure. The fit of the models was assessed by the Hosmer-Lemeshow goodness-of-fit test. The models were considered acceptable if p was > 0.05, and all models met this demand [17]. The statistical power was 82%, which is acceptable.

### Ethical Considerations

The study was approved by the Regional Ethical Committee of the Karolinska Institute (reference no. 2006/4:8, 2006-09-27).

## Results

### Sociodemographic background

In total, we examined 173 Assyrian/Syrian patients along with 181 native Swedish patients. The proportions of men and women were similar (table 1). The Swedish population was somewhat older with a mean age of 64 years and the range was 32-86 years, while the Assyrian/Syrian population was younger with a mean age of 61 years and the range was 32-83 years. For example, about 31.5% of the Swedish subjects were in the age group > 70 years, while only 23.7% of the Assyrian/Syrians were in that group. Furthermore, there were socioeconomic differences between these two groups. Assyrians/Syrians had a lower educational level (20% of them were illiterate [25% of women and 14.6% of men]). Compared to only 12.1% of Assyrian/Syrian subjects, 37.1% of Swedes subjects lived alone, while 78.6% of Assyrians/Syrians were married compared to 60.1% of native Swedish subjects. The proportion of employed individuals owning housing was much higher in the Swedish ethnic group: 57.3% compared to 14.5% among Assyrians/Syrians.

**Table 1:** Distribution (%) and anthropometric data (means; standard deviations) of background variables in Assyrian/Syrians and Swedish patients, n = 354.

Sociodemographic variables	Assyrians/Syrians n = 173	Swedes n = 181	Test of difference P-value
Total	48.9	51.1	
**Gender**			
Female	48.5	44.2	0.411
Male	51.5	55.8	
			
**Age (years)**			
32-57	39.3	27.1	0.003
58-70	37.0	41.4	
> 70	23.7	31.5	
			
**Housing tenure**			
Rental	85.5	42.7	0.000
Ownership	14.5	57.3	
			
**Educational status**			
Low < 9 years	74.0	49.1	0.000
Intermediate 9-11 years	4.6	20.9	
High > 11 years	21.4	30.0	
			
**Employment status**			
Yes	18.9	32.0	0.006
No	81.1	68.0	
			
**Anthropometric data**			
Weight (kg)	82 (17.5)	87 (19.1)	0.012
Height (cm)	162 (9.9)	170 (9.6)	0.000
BMI (kg/m^2^)	33 (20.2)	30 (6.0)	0.059

### Cardiovascular risk factors

The prevalences of increased blood glucose, blood lipids, hypertension and increased albumin are shown in table 2. The prevalence is defined in terms of individuals with non-normal measurements. There were no significant differences in the HbA1c level and total cholesterol between Assyrians/Syrians and Swedes. The prevalence of urinary albumin was non-significant (43.2% vs 38.1%). On the other hand, Swedes had a significantly higher prevalence of hypertension (76.8% vs 57.8%) and of increased triglycerides (61.5% vs 39.7%) than Assyrians/Syrians.

**Table 2:** Prevalence (%) and means (standard deviations) of the outcome variables increased HbA1c, increased blood lipids (triglycerides and total cholesterol) and hypertension (systolic/diastolic) in Assyrian/Syrian and Swedish type 2 diabetes patients by ethnicity.

Variables	HbA1c (>6%)		Triglycerides (≥ 1.7 mmol/L)		Total cholesterol (≥ 4.5 mmol/L)		Hypertension (systolic>130 mm Hg or diastolic >80 mm Hg)	
	**Swedish**	**Assyrian**	**Swedish**	**Assyrian**	**Swedish**	**Assyrian**	**Swedish**	**Assyrian**

**Mean**	6.1 (1.2)	6.3 (1.6)	2.1 (1.0)	1.7 (1.0)	5.0 (1.1)	4.8 (0.9)	136.2 (17.3)/78.5 (11.3)	137.0 (15.6)/78.6 (9.1)
								
**Totals**	40.6	49.7	61.5	39.7	66.5	58.8	76.8	57.8
								
**P-value**			***				***	
								
**Age**								
32-57	48.9	55.2	57.5	37.7	63.4	55.4	73.5	44.1
P-value			**				**	
58-70	37.5	42.2	66.2	37.5	60.3	61.3	75.0	62.5
P-value			**					
> 70	37.5	52.5	58.1	48.3	78.3	62.5	82.5	72.5
P-value								
								
**Gender**								
Female	46.8	52.4	68.3	40.0	70.8	65.4	76.3	54.2
P-value			**				**	
Male	35.7	47.2	56.5	39.5	63.3	53.1	77.2	60.7
P-value			*				*	
								
**Housing**								
Ownership	37.9	48.0	59.2	30.0	67.1	43.5	80.6	48.0
P-value			*		*		**	
Rental	47.1	50.0	64.5	41.3	63.5	61.8	74.0	59.2
P-value			**				*	
								
**Education**								
Low	37.9	50.8	54.3	40.6	62.7	62.1	74.2	64.1
Intermediate	46.0	62.5	76.5	28.6	70.6	62.5	84.2	12.5
P-value			*				***	
High	41.2	42.9	61.4	39.4	69.6	48.6	75.9	44.4
P-value			*		*		**	
								
**Employment**								
Yes	40.0	48.4	62.0	29.0	67.3	45.2	76.0	45.2
P-value			**		*		**	
No	40.8	50.0	61.2	42.6	66.0	62.5	77.0	60.3
P-value			**				**	

P-value are presented as * p < 0.05; ** p < 0.01; *** p < 0.001

The prevalence of the outcome variables by explanatory variables and ethnicity are also shown in table 2. The prevalence of increased triglycerides was higher among Swedish-born individuals in age groups under 70 with intermediate and high educational levels. We found significant ethnic differences in hypertension: in Swedish-born individuals in the age group 32-57, in intermediate and highly educated Swedish-born subjects. The prevalence of increased total cholesterol in Swedish-born employed subjects owning housing and with intermediate and high educational levels was significantly higher than in Assyrian/Syrians.

### Logistic regression

**Figure 1:**
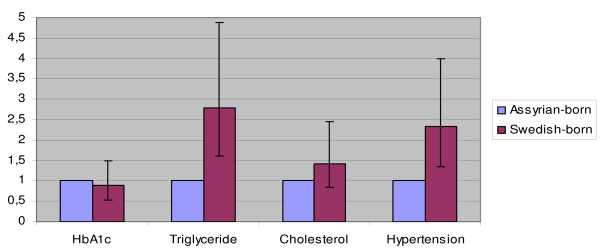
**Final models**. The odds ratios (ORs) with 95% confidence intervals (CI 95%) for increased hbA1c (M 1), for increased triglycerides (M 2), for increased total cholesterol (M 3) and hypertension (M 4) in Swedish-born subjects and Assyrians/Syrians as the reference group after adjusting for explanatory variables.

The odds ratios in the crude logistic regression models for hypertension and increased triglycerides were about two and half times higher among Swedish-born subjects than among Assyrians/Syrians (OR = 2.42; 95% CI = 1.5-3.8 and OR = 2.45; 95% CI = 1.5-3.9 respectively) (not shown in table). Crude odds ratios for having increased levels of HbA1c and total cholesterol were similar among Swedish-born individuals and Assyrians/Syrians. The results of the final logistic regression models (M1-M4) are shown in table 3 and in figure 1 for the association between ethnicity and four cardiovascular risk factors, adjusted for age, gender, employment and housing tenure. Model 1 shows that there were no significant ethnic differences in odds for having higher levels of HbA1c (< 6%) between Swedish-born and Assyrian/Syrian-born individuals. In model 2, Swedish-born patients have higher odds for increased triglycerides (OR = 2.80; 95% CI = 1.61- 4.87) than Assyrian/Syrian-born patients. There were no differences between the two ethnic groups regarding high cholesterol levels. On the other hand, the odds for having hypertension were more than doubled in Swedish-born subjects than in Assyrian/Syrians (OR = 2.32; 95% CI = 1.35- 4.00). Women have higher odds for increased total cholesterol than men (OR = 1.6; 95% CI = 1.01-2.64). Stepwise introduction of employment status in the model decreased odds ratios for having hypertension in Swedes to OR = 2.20 (95% CI = 1.36-3.58) and increased very slightly to OR = 2.32 (95% CI = 1.35-4.00) when the variable housing tenure was added in the final model (not shown in table). Age and gender-adjusted odds ratios for having abnormal values in triglycerides levels in Swedish-born individuals decreased to OR = 2.40 (95% CI = 1.50-3.90). Inclusion of employment status in the model increased the odds ratio to OR = 2.48 (95% CI = 1.52-4.10) and housing tenure to OR = 2.80 (95% CI = 1.60-4.87). The ethnic differences in hypertension and increased triglycerides between Swedish-born and Assyrian/Syrian subjects remained significant after adjusting for the explanatory variables.

**Table 3:** The odds ratios (ORs) with 95% confidence intervals (CI 95%) for increased hbA1c (M 1), for increased triglycerides (M 2), for increased total cholesterol (M 3) and hypertension (M 4) in Swedish-born subjects and Assyrians/Syrians as the reference group after adjusting for explanatory variables.

Variable	Model 1 (HbA1c)	Model 2 (triglyceride)	Model 3 (cholesterol)	Model 4 (hypertension)
**Assyrians/Syrians**	**1**	**1**	**1**	**1**
**Swedes**	0.90 0.54-1.48	2.80 1.61-4.87	1.42 0.83-2.44	2.32 1.35-4.00
				
**Gender**				
Male	**1**	**1**	**1**	**1**
Female	1.40 0.90-2.14	1.30 0.80-2.11	1.64 1.01-2.64	0.82 0.51-1.32
				
**Age groups (years)**				
32-57	**1**	**1**	**1**	**1**
58-70	1.19 0.69-2.01	1.19 0.69-2.10	1.19 0.66-2.17	1.20 0.65-2.21
> 70	1.21 0.64-2.27	1.20 0.64-2.27	0.97 0.45-2.06	1.10 0.49-2.28
				
**Housing tenure**				
Ownership	**1**	**1**	**1**	**1**
Rental	1.30 0.76-2.13	1.33 0.75-2.38	1.17 0.67-2.04	1.00 0.65-1.77
				
**Employment**				
Yes	**1**	**1**	**1**	**1**
No	0.82 0.45-1.50	0.89 0.47-1.68	0.92 0.50-1.70	1.10 0.59-2.10

## Discussion

The main finding of this study is the differences between ethnic Swedes and Assyrians/Syrians with type 2 diabetes in increased levels of serum triglycerides and hypertension. Ethnic Swedes had twice higher odds of increased serum triglycerides than Assyrian/Syrians. The same pattern was observed regarding hypertension.

The prevalence of type 2 diabetes in Assyrians/Syrians in the present study agrees with a number of studies in immigrant populations in general [18] and in particular with studies from the Middle East that have shown than these subjects have a higher prevalence of diabetes than the native population [6,8,11,19-21]. A review of 18 studies on Turkish and Moroccan immigrants in Northwestern Europe found a two to four times higher prevalence of type 2 diabetes in both groups compared to a Dutch population [22] and lower cardiovascular mortality rates in Turkish immigrants, and the absolute risk for a coronary event in this group was similar to the risk in native Dutch diabetes patients [14]. Because of a lack of valid studies on blood pressure and lipids in this review, we cannot compare our results. In the present study we have contributory findings on higher risks for a coronary event in Assyrians/Syrians than in native Swedes because of elevated triglycerides levels.

It is also a well-known fact that elevated triglyceride levels contribute to an increased risk of cardiovascular disease independently and, in combination with, among other factors, obesity, metabolic syndrome and type 2 diabetes affect the cardiovascular disease risk [23,24]. Elevated triglyceride levels have been shown to be more prevalent in urban populations [25,26]. The population in the present study is an urban one with more disadvantaged socioeconomic conditions in Assyrians/Syrians than in native Swedes, but despite this fact we found higher increased triglyceride levels in the indigenous population than in immigrants.

Available data from 2007 on the general Swedish population in the National Diabetes Register (NDR) [5] make it possible to compare our results with countrywide results: the prevalence of normal HbA1c levels in the Swedish-born subjects in the present study is slightly higher than in the Swedish population in general (59.4% vs 58.1%). The prevalence of normal HbA1c in Assyrians/Syrians is somewhat lower than in the general population (50.6% vs 58.1%). The normal level of total cholesterol in the sample in this study is lower than in the general Swedish population according to the NDR (33.5% vs 43.1%), but it is almost on the same level in Assyrian/Syrian subjects (41.2% vs 43.1%). According to the NDR, 55.5% of the general population reach the target for an acceptable level of triglycerides (< 1.7 mmol/L), which is quite similar to our results in Swedish-born subjects (49.5%) and lower than in Assyrian/Syrian ethnic group (61.3%). The number of individuals with a normal blood pressure (≤ 130/80 mmHg) in the general population is higher than in the Swedish sample in the present study (35% vs 23.2%) and lower than in Assyrians/Syrians (35% vs 42.1%).

Our findings of ethnic differences in lipids are in accord with the results of other studies, e.g. high cholesterol levels were examined in an Swedish study and were found to be associated with younger older and longer education in a primary-health-care-based programme for cardiovascular prevention in both native-born and foreign-born individuals. However, no significant predictor was detected for the reduction of high triglyceride levels [27].

Our finding of an increased prevalence of hypertension in Swedish-born subjects contrasts with a large number of studies comparing immigrant groups with the host population. For example, in a meta-analysis of 125 studies investigating an association between acculturation and blood pressure, the investigators concluded that there were changes in blood pressure due to acculturation to Western society and that this was not related to body mass index (BMI) or cholesterol. It was concluded that the higher blood pressure in immigrants was associated with acculturation, with the stress of cultural change being the major component. Despite a lack of information as to whether the participants in those studies had diabetes or no, it is interesting to compare to the results in the present study, with the host population having a significantly higher prevalence of hypertension than Assyrian/Syrian patients with type 2 diabetes.

Studies done in Sweden have shown that immigrants perceived their health to be poorer, and they have onsets of disease earlier and are more overweight than native Swedish individuals with type 2 diabetes [6,28]. The following factors pertaining to migration itself and to the lifestyle after migration have been cited as explanations: low levels of employment and education and a higher prevalence of overweight and obesity among immigrants in general and in women from Turkey in particular [11]. The focus of the present study was not on investigating the onset of diabetes or the prevalence of overweight, but the results do not agree with the findings of studies documenting poor health in immigrants as compared to native-born populations [29-32].

It is a known fact that food intake, especially increased carbohydrate intake, is a common reason for elevated triglycerides [24]. It is possible that the Mediterranean diet [33] of Assyrians/Syrians decreased the risk for higher levels of lipids, but we have no data concerning this in the present study and the matter should be investigated in prospective studies.

The major strength of this study is that this is the first survey describing a somewhat homogeneous Assyrian/Syrian ethnic group identifying themselves in terms of their actual ethnicity and not of a classification based on country of birth or regional and geographic affiliations. Despite the small sample size, the study may be considered to be representative of Assyrians/Syrians from four countries living in Sweden. Even if we cannot generalise our findings to all Assyrian/Syrian and Swedish-born patients, the results of the study provide a reliable baseline for future studies.

A major limitation of the study is its cross-sectional nature and that the small number of participants precludes the possibility of drawing extensive causal conclusions. We are not able to estimate the number of Assyrians/Syrians ethnic group in Sweden because they are registered as citizens of the countries from which they come, but they originate predominantly from Turkey and so is their representation in our study with 33.5% coming from that country.

## Conclusion

The results of this study show that ethnic differences regarding triglycerides and hypertension between Assyrian/Syrian and Swedish type 2 diabetes patients could not be explained by differences in the sociodemographic situation but might have other explanations such as differences in biological or lifestyle factors. Nevertheless, the findings of this study highlight the importance of the lipid control and reaching the targets for normal levels in subjects having diabetes with the purpose of minimising the cardiovascular risk.

## Abbreviations

CI: confidence interval; OR: odds ratio.

## Competing interests

The authors declare that they have no competing interests.

## Authors' contributions

MT conceived the idea of the study and carried out the data collection. JT, AW and MT designed the study. SEJ and MT performed the statistical analysis. JS and SEJ drafted the manuscript. JS, JT, AW and MT revised the manuscript. All authors read and approved the final manuscript.

## Author information

MT has a PhD degree in Medical Science, AW is an assistant professor, SEJ is a professor, JT is a GP with MD and PhD degrees and JS is a professor.
